# The evolution of percutaneous abdominal abscess drainage: A review

**DOI:** 10.1097/MD.0000000000041799

**Published:** 2025-04-11

**Authors:** Christopher Stevens, Dylan Scott, Prerana Ramesh, Amanda Ragland, Coplen Johnson, Joshua Strobel, Kevin Malone, Horacio D’Agostino, Chaitanya Ahuja, Luis De Alba

**Affiliations:** a School of Medicine, LSU Health Shreveport, Shreveport, LA; b Department of Radiology, LSU Health Shreveport, Shreveport, LA; c Department of Emergency Medicine, Baylor Scott and White Medical Center, Temple, TX.

**Keywords:** computed tomography, history, percutaneous abscess drainage, ultrasound

## Abstract

Abdominal abscesses are commonly treated using percutaneous abscesses drainage (PAD). Despite PAD becoming commonplace in clinical practice, there are still unanswered questions and absent formal guidelines regarding its use. This narrative review discusses the evolution and different features of PAD, including the imaging modalities used in the process, catheter anatomy and sizes, placement techniques, the quantity of catheter side holes, and factors that determine the length of catheter stay and the usage of antibiotics for treating abdominal abscesses. Based on a retrospective review of the existing literature, this work is presented with an intent of identifying the most efficient design aspects and indications to optimize clinical success when performing PAD. This manuscript shows that there are still many unanswered questions and room for improvement regarding percutaneous drainage of abscesses.

## 1. Introduction

An abscess is described as a collection of purulent material that usually forms from infection-induced inflammation. Most abscesses, no matter the location, require drainage.^[1]^ Image-guided percutaneous abscess drainage (PAD) using either computed tomography (CT) or ultrasound (US), is a minimally invasive procedure performed commonly by interventional radiologists that involves deploying a drainage catheter into the abscess by perforating the skin and overlying soft tissues. Abdominal abscesses are commonly treated with PAD.^[2]^ Compared to surgical drainage, PAD is associated with a higher success rate, faster recovery time, decreased morbidity, mortality, and cost.^[3,4]^ Despite the increase in usage of PAD for abdominal abscesses, there are still some unanswered questions and absent guidelines regarding its use; herein, we present an updated review on PAD while also reviewing the evolution of this procedure.

## 2. Methodology

A literature search was conducted using PubMed, EBSCO, and Google Scholar databases in December 2023. The literature was reviewed to identify articles focused on the evolution and features of percutaneous abscess drainage and to investigate the most up-to-date imaging modalities, catheter anatomy, catheter placement, catheter sizing, quantity of catheter side holes, length of catheter stay, and use of antibiotics in patient care. A search string using the following Medical Subject Headings (MeSH): “percutaneous catheter,” “abdominal abscess drainage,” was used to identify the most relevant articles. This review was compiled in accordance with the Scale for the Assessment of Narrative Review Articles (SANRA) tool. Specific inclusion criteria included articles written in English that discussed the use of percutaneous drainage catheters and/or the treatment of abdominal abscesses. Additionally, a literature review of the emerging technologies surrounding this procedure was performed using the same databases. Using a search string of the following MeSH was used for the literature review involving the role of artificial intelligence (AI) in PAD: “AI,” “robot-assisted,” “machine-learning.”

The goal of this data collection was to create a comprehensive literature review of the above-mentioned aspects of catheter-guided percutaneous abdominal abscess drainage, from the historical development to the best clinical considerations regarding the usage of this procedure.

## 3. Review

### 3.1. History of CT and US

Being that CT and US have such a valuable impact on how we perform PAD procedures, it would be dishonorable to not discuss the history of these imaging modalities and show respect to the pioneers of them in this review.

The initial idea of CT can be traced back to 2 early papers published by a physicist named Allen Cormack (1924–1998) in 1963 and 1964.^[5]^ He first proposed a method to improve upon the concept of tomography by measuring X-rays once they have passed through the body to calculate the amount of radiation that had been absorbed.^[6]^ In 1964, he proposed mathematical formulas for constructing cross-sectional images using the measurements.^[7]^ Though these published articles generated little interest from the medical field at the time, the true significance of Cormack work was eventually recognized, which resulted in him sharing the 1979 Nobel Prize for Physiology of Medicine with Sir Godfrey Hounsfield.^[5]^

By 1968, Sir Godfrey Hounsfield (1919–2004) proposed an idea to enhance tomographic X-ray images by using computers to recognize and display patterns of numerical data.^[8]^ Although Hounsfield did not know about the previous papers published by Cormack, their ideas were similar. His proposal was approved in 1968 and he began building rudimentary prototypes with the help of funding from the British Department of Health and Social Services.^[8]^ Hounsfield, along with the help of neurologist Dr James Ambrose, performed the first CT scan using his prototype on October 1, 1971, at Atkinson Morley hospital in London, England.^[9]^ The scan took place on 1 of Dr Ambrose patients who was suspected to have a tumor within her frontal lobe. The original scan produced an image with an 80 × 80 matrix and took 5 minutes per scan and another 5 minutes to process the image data. The surgeon who later removed the tumor from the patient reported that the tumor looked identical to the 1 seen in the image of the scan.^[10]^

**Figure 1.**
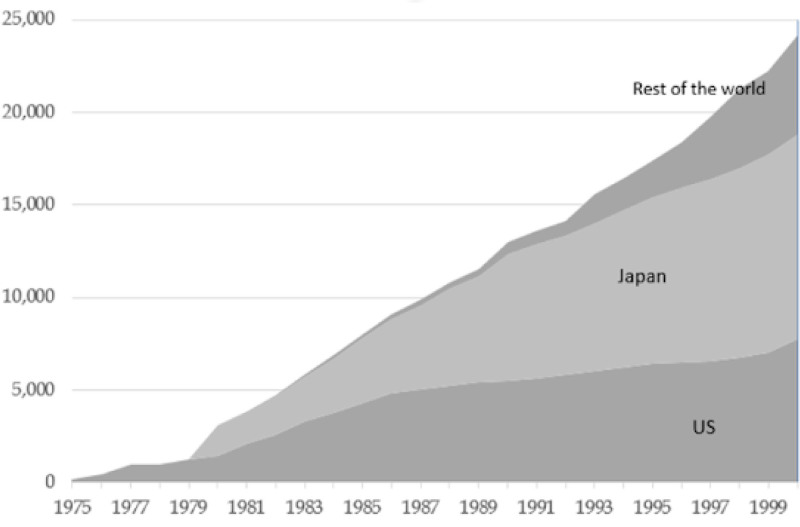
Number of CT units installed in U.S., Japan, and rest of the world from 1975 to 2000. © 2018 Bhidé, Datar, & Stebbins.^[8]^ Licensed under the terms of the Creative Commons Attribution 4.0 International License (https://creativecommons.org/licenses/by/4.0/).

The use of CT scan has risen since its implementation, with the United States and Japan leading the way due to more relaxed regulations that decreased the fees for CT scans and offered more scanners in more remote and smaller health institutions (Fig. 1).^[8]^ Improvement in CT technology also accompanied the growing rate of CT use. Advances in CT mainly targeted the areas that early imaging was weak in, such as image acquisition and processing speed, resolution, and machine specific metrics, such as rotations of the aperture or detectors within the machine. Given that CT imaging is a source of ionizing radiation, improvements in the time spent in the scanner would ideally be minimalized while maximizing the resolution at which the images are captured at. Since the last half of the 20th century, minimum scan time has decreased dramatically.^[11]^

Diagnostic US is an imaging modality that utilizes sound waves to create images of internal body structures. Due to its ease of use, reliability, low cost, limited risk association, high degree of mobility, and no radiation emission, it has become 1 of the most popular medical imaging techniques used for point-of-care applications in hospitals worldwide.^[12]^ Not only is this beneficial for critically ill patients who may not have access to other routinely used imaging modalities, but it also allows for quicker and more direct decisions to be made by the physician since the ultrasound images are collected instantly.^[13]^

The development and evolution of ultrasound into its contemporary iteration came from contributions of many different people in the scientific community, including physiologists, physicists, electrical engineers, neurologists, and obstetricians. Perhaps 1 of the most fascinating parts of its history is that it theoretically started with the study of bats in 1793.^[14]^ Lazzaro Spallanzani (1729–1799), an Italian physiologist and priest, experimented with bats and found that when they were blinded, they could still fly through space accordingly. However, when the bats’ sense of hearing was deceased, they could no longer fly safely. From this observation, Spallanzani concluded that bats relied on sound waves to navigate, the same wave type utilized by ultrasound machines.^[15]^ Approximately 50 years after Spallanzani findings, the first postulation of the Doppler effect, a concept that describes how the frequency of light and sound waves become altered when there is motion the source or sensor emitting the waves, was made by an Austrian physicist named Christian Doppler (1803–1853).^[16]^

In addition to bats and trumpets, submarines also possess a place on the timeline of ultrasound development. As German submarines became a threat to Allied ships during War World I, Paul Langevin (1872–1946), a French physicist, and his colleagues were ordered by the French government to figure out a way to detect German submarines using high-frequency sound waves.^[17]^ This led to the construction of an underwater sandwich sound generator that was able to detect the returning echoes from submerged submarines.^[18]^ This technology was improved throughout World War I and World War II, eventually leading to the development of medical diagnostic ultrasound.^[15]^ The first ever usage of ultrasound as a medical diagnostic tool occurred in 1942 when an Austrian Neurologist named Karl Dussik used it to locate brain tumors and the cerebral ventricles.^[19]^ This historic event was then followed by a fascinating experiment in 1949 carried out by an American named George Ludwig (1922–1973) at the Naval Military Research Institute in the United States. Ludwig inserted human gallstones into multiple dogs and subsequently was able to show echoes that were consistent with the implanted gallstones, thus making it the first in vivo depiction of human gallstones in an animal model.^[17]^ While this was an important milestone in the development of ultrasound technology, perhaps the most defining moment occurred in 1956 to 1957. During this time, a European obstetrician named Ian Donald continued to improve on the ultrasound diagnostic techniques, ultimately leading to the construction of an ultrasound scanner mounted to a ceiling that could image an object underneath. He then used this technology to diagnose an ovarian cyst.^[17]^ While the quality of images produced by ultrasound was still poor at this time compared to today’s standard, the successful work of these pioneers should not be described as anything other than extraordinary as their work and commitment resulted in the development of an imaging modality that revolutionized the healthcare world.

### 3.2. Use of CT and US in PAD

CT and US are commonly used imaging modalities to guide catheter placement for PAD. Both image-guided techniques are alternatives for surgical management of abscesses and possess a shorter recovery time compared to laparoscopic options.^[2]^ In addition, these procedures avoid general anesthesia, result in lower morbidity, and have a lower cost compared to surgical drainage.^[20]^ CT-guided PAD is preferred when visceral, vascular, and skeletal structures have the potential to interfere with imaging; in addition, it is also preferred in obese patients.^[2]^ Compared to US-guided PAD, CT-guided PAD is less operator-dependent and can ensure that the patient remains in a stable position^[2]^; however, US-guidance has several advantages. Three major ones are that it offers a real time view, has no radiation-exposure, and expedites treatment times in comparison to the wait times for a CT machine to be available.^[2]^

### 3.3. Brief overview of abscess formation and diagnosis

An abscess is defined as a collection of pus and is usually caused by a bacterial infection, most commonly *Staphylococcus aureus*. The most common sites for abscesses to form are skin and soft tissues, although they have the potential to form anywhere in the body where microorganisms have access.^[4]^ The pathophysiological process of abscess formation is initiated once the body develops an infection, and a localized acute inflammatory response begins to “wall off” the infection. While this step of “walling off” the infection is important in limiting the dissemination of the microorganism, it also allows for an inflammatory exudate to form in the center that is filled with polymorphonuclear leukocytes, tissue debris, fibrin, and live bacteria.^[21]^ As the abscess matures, fibroblastic proliferation occurs and there is formation of a peripheral fibrous capsule.^[21]^ This process eventually leads to patients experiencing fever, chills, and pain in the approximate location of the abscess. Diagnosis of an abscess is usually made by combination of clinical examination and medical imaging.

### 3.4. The evolution of catheter use and development

The word “catheter” is derived from the Greek word καθίημι (kathíēmi), meaning “to let down or descend.” In simple terms, a catheter is a piece of tubing inserted into the body - through natural orifices or percutaneously – to create an opening that allows 1 to administer a drug, distend a passageway, or remove fluid,^[22]^ with the latter being the focus of the present article. One of the earliest records of catheters use can be dated back to approximately 1500 BCE, when Egyptians used drainage catheters made from straw, reeds, curled up palm leaves, and bronze tubes to relieve urinary retention in males. Years later, around 400 BC, Hippocrates of Kos (c. 460 - c. 375 BCE) refers to the use of lead tubes as a method of alleviating urinary retention.^[23,24]^ Hippocrates advocated for the use of chest tube drainages, describing the treatment of empyema by means of insertion of metal tubes.^[25]^ Around AD 100, Claudis Galen mentioned the use of nonflexible catheters to irrigate the bladder in both males and females as well as the irrigation of ears.^[26]^ Records regarding catheter use were relatively stagnant until about the 1100s when the Chinese described the treatment of urinary retention by transurethral insertion of hollow leaves of onion.^[23]^

Aside from the design of the catheter itself, how catheterization procedures are performed has evolved throughout history as well. During the Middle Ages, these procedures were commonly performed on patients while they remained in an upright, kneeling, or seated position.^[26]^ Today, catheterization procedures are routinely performed on patients while they are lying down on a table.

It was not until around 1850 that the first flexible rubber catheter with a solid tip made of vulcanized rubber was used by Auguste Nélaton (1807–1873).^[27]^ This was a landmark advancement to the field of medical instrumentation leading to further development of catheters. However, easy catheter dislodgment in the presence of minimal traction was a major drawback. External fixation of the Nelaton catheter with adhesive tape or a skin suture were insufficient to prevent spontaneous catheter removal.^[23]^ This shortcoming stimulated the development of internal retention mechanisms, such as mushroom tips (Malecot, Pezzer), that had partial success in securing urinary catheters. The Foley catheter, a urinary drainage catheter with a balloon retention device, was developed in the 1929 and became the standard for bladder catheterization.^[28]^

Catheter drainage of fluid collections had an early surgical stage until the late half of the 1900s when collections were evacuated by placing catheters intraoperatively without imaging. The approaches included a simple incision and drainage for superficial collections or open surgery for deep seated abscesses. The “minimally invasive drainage” concept was described in the 1950s by Debakey and Welch.^[29]^ The authors provided evidence on the effectiveness of subphrenic and other locations of abscesses evacuated by needle aspiration or placement of a catheter within the abscess or collection during an operation. McFadzean et al in 1953 published on “intraoperative aspiration of liver abscesses with needle and antibiotics” as a curative procedure in 14 patients.^[30]^ This was initially harshly challenged in the surgical literature as an unsafe procedure bound for severe outcomes. However, aspiration of most of the purulent material combined with antibiotics certainly worked for liver abscesses.^[29,30]^

The use of US-guidance for percutaneous drainage was reported in the 1970s.^[29,31]^ It was, however, the emergence of CT that truly stimulated percutaneous drainage of fluid collections in America.^[29]^ Numerous articles were published in the 1980s establishing the techniques, results, complications, and controversies of percutaneous drainage, notably by Gerzof,^[32]^ Haaga,^[33]^ Karlson,^[34]^ Martin,^[35]^ vanSonnenberg,^[36]^ among many others. From this point and based on these early pioneers’ work, indications and image-guided drainage catheter technique evolved and expanded.

### 3.5. Basics of catheter anatomy

**Figure 2.**
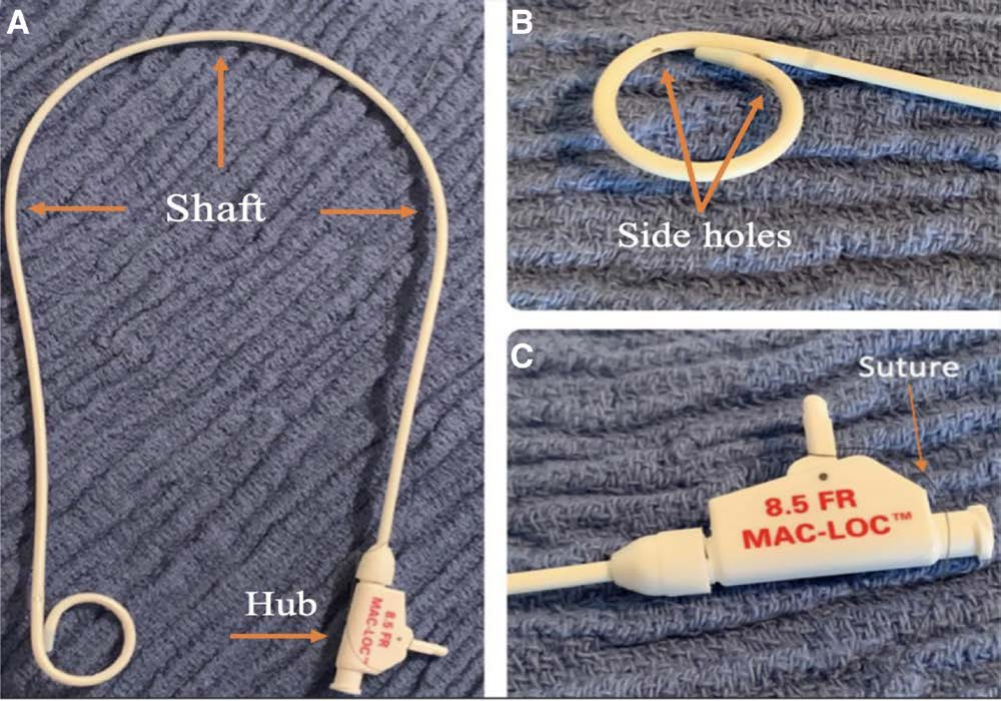
Images depicting the anatomy of a pigtail cope loop catheter. (A) Whole pigtail cope loop catheter. (B) Distal end of pigtail cope loop catheter (resides in a pigtail loop which aids in fixation of the catheter). (C) Proximal end of pigtail cope loop catheter (contains the suture that is pulled to create the pigtail loop at the distal.

**Figure 3.**
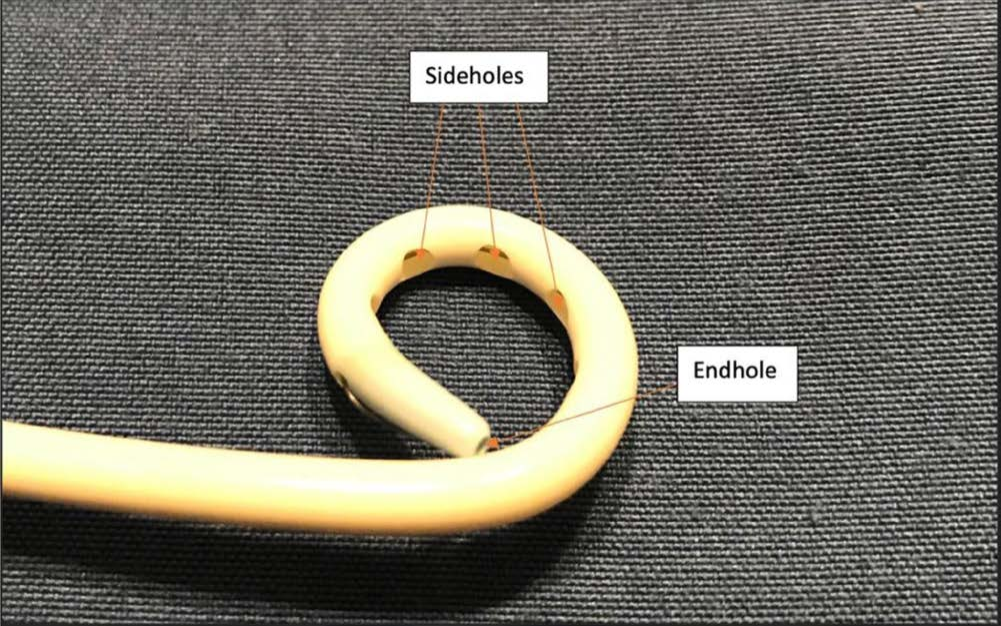
Image of the distal end of a pigtail drainage catheter with side holes and an end hole.

Figures 2 and 3 depict the basic anatomy of a pigtail catheter, a catheter type that is commonly used in drainage of fluid collections. The proximal end of the catheter contains the catheter hub, the section that houses the part of the suture that is pulled once the catheter is in place, thus allowing for formation of the eponymous pigtail loop at the distal end which aids in catheter stability. The proximal end is also the part of the catheter that is attached to a collection bag that the drained fluid resides in once removed from the body. Between the proximal and distal end of the catheter is the shaft. The distal end of the catheter houses the side holes and end hole, which is placed inside the abscess.

The catheter shaft has an outer diameter and inner lumen diameter. The size of the catheter corresponds to the measurement of the outer diameter of the catheter shaft. The French (F) scale, invented in 1842 by a Parisian named Joseph-Frédéric-Benoît Charrière (1803–1876),^[37]^ is used for sizing the outer diameter of drainage catheters, which may range from 6 to 32 F dependent on the patient and fluid collection characteristics (Table 1).

**Table 1:** Relationship between outside diameter in millimeters of catheter and French size of catheter.

OD of catheter (mm)	French size
2.67	8F
3.33	10F
4.00	12F
4.67	14F
5.33	16F
6.00	18F
6.67	20F
7.33	22F
8.00	24F
8.67	26F
9.33	28F

1F = 0.33 (1/3) mm.

F = French, OD = outside diameter.

### 3.6. How is a catheter placed?

After assessment of the patient’s abscess via medical imaging, the interventional radiologist or primary operator can choose the technique for the best way to access the abscess and drain it. The Seldinger and Trocar techniques are routinely used for percutaneous drainage catheter insertion.^[2]^ The Seldinger technique was introduced in 1953 when Sven–Ivar Seldinger (1921–1998) published descriptions of his revolutionizing technique in Acta Radiologica and is widely applied for catheter delivery in vascular and nonvascular procedures today.^[38]^ This method involves using a needle to first puncture the skin and gain sight to the target area, followed by insertion of a guidewire through the needle, then removal of the needle and threading of the catheter over the guidewire followed by guidewire removal. Before Seldinger findings, physicians wanting to drain an abscess or gain vessel entry used a large bore needle in which a narrower bore catheter was then threaded through it. Compared to Seldinger technique, downfalls to this method was that it restricted its use to larger vessels, made vessel puncture more difficult, and increased the risk of hemorrhagic occurrence.^[39]^ The special piece of Seldinger method that made it more efficient compared to earlier techniques was the use of the guidewire, a flexible, round-ended, metal leader. The introduction of this piece allowed for insertion of a catheter that housed the same bore as the needle instead of having to use a larger bore needle.^[39]^

The Trocar technique is primarily recommended for larger and more superficial collections.^[40]^ Compared to the Seldinger technique, which is a 2-step process, the Trocar technique is a 1-step process that involves advancing the catheter to the target location with a metallic stiffener and Trocar needle that are loaded inside the catheter lumen. After imaging confirms that the device is in the correct area, the catheter is placed by releasing it over the stiffener. While this method is known to be quicker compared to the Seldinger technique, its disadvantages include increased pain to patient, greater morbidity, and increased risk of adjacent organ damage and hemorrhage.^[40]^

### 3.7. How many side holes should catheters have?

**Figure 4.**
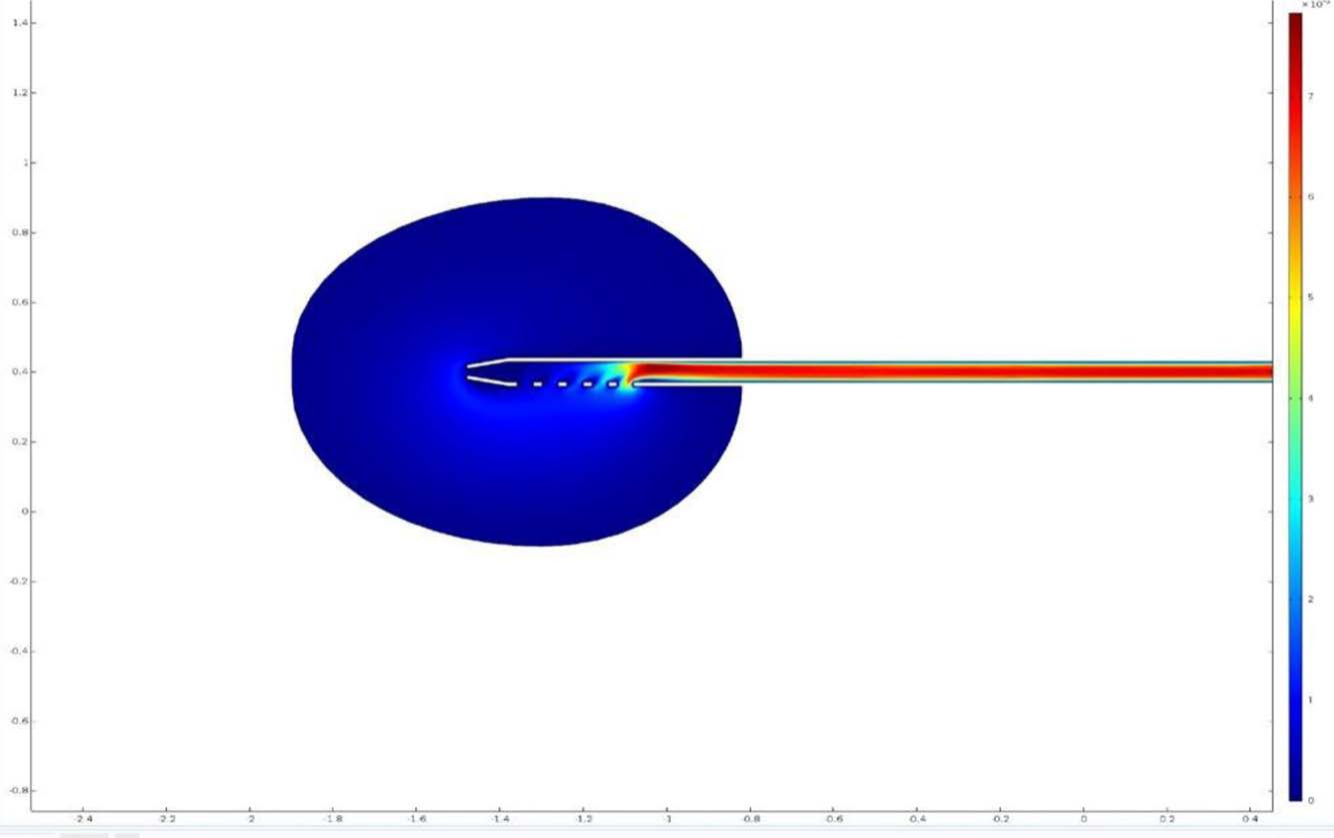
A 2D computational model demonstrating the flow characteristics of a catheter with 5 unilateral side holes and 1 distal end hole. The flow rapidly decreases in distally located side holes with stagnation of flow observed proximally to the end hole.

Despite the pervasiveness of PAD, there has been a general lack of emphasis regarding the quality and functional aspects of current commercial drainage catheters – particularly the number of side holes present on catheters. Many companies produce catheters with >5 side holes and propose that catheters with more side holes produce better drainage efficiency, but the evidence disagrees. Ballard et al found that most drainage occurred at the most proximal side holes, with a 3 side hole catheter producing the most drainage efficiency.^[41]^ Similarly, Schwartz and Vaughan-Neil evaluated the performance of pigtail catheters with varying number of side holes by looking at quality of left ventricular opacification, lack of catheter recoil during angiography, and absence of complications. Outcomes showed that catheters with fewer side holes not only performed similarly catheters with a greater number of side holes, but also decreased the risk of thromboembolism associated with pigtail catheter usage.^[42]^ Muath Bisawi et al utilized an in-silico simulation to compare efficiency of straight and pigtail catheters and concluded that most flow in both catheter types occurred at the most proximal 1 to 3 side holes, with relatively little flow occurring at more distally located side holes (Fig. 4).^[43]^ Pope et al took a different approach and utilized an in vivo design to examine how contrast exited side holes of pigtail and biliary catheters in 99 patients. This study showed that the most proximal side holes contributed a greater percentage of drainage potential than the distal side holes.^[44]^ In summary, most commercially available catheters are configured with greater than 6 side holes; and while seemingly clinically effective, this catheter design is not optimal according to the literature.

### 3.8. What size catheter to use for drainage of an abscess?

The choice of catheter size is an example of how drainage of an abscess can differ among patients, clinicians, and institutions. The physician will determine the catheter size at his or her discretion based on a variety of considerations, including the need of the patient, characteristics of the abscess as seen on prior imaging, and nature of the abscess fluid including viscosity.^[45]^ The range of catheters for operators to choose for abscess drainage is extensive: non-sump catheters from 6 to 12F in diameter, sump drains with double lumen from 12 to 18F size, Malecot catheters of many sizes, and Trocar catheters for single-step drainage.^[46]^ Currently, there is an absence of standardized guidelines on which exact size catheter to use for drainage of an abscess; choice is currently made based on personal preference and user experience.^[47]^ Larger catheter sizes are commonly recommended and appropriate for effective drainage of thick, viscous abscess contents.^[46]^ A recent study conducted by Celik, et al concluded that 8F catchers were appropriate for most percutaneous drainages of body fluid collections.^[47]^ It has been reported that operators will elect for a larger size catheter in the 10 to 14F range because it is frequent to have to replace an 8F catheter due to insufficient drainage^[46]^; however, the larger size catheters present challenges and disadvantages as well, including an increase in patient discomfort and procedure time, higher risk of complication and increased bleeding.^[47]^ Park et al found that larger catheters provided faster drainage of the abscess, but the clinical outcome was not necessarily better.^[48]^ Smaller catheters allow for more patient comfort, shorter procedure times, and lower rates of post-procedural complications.^[47]^ Alternatively, some studies have reported that a difference in catheter size does not result in altered drainage time, complication rates, recurrence rates, or hospital stays.^[46]^ More research is warranted to better define guidelines for choosing which catheter size is best for PAD as the current variability in catheter size adds variability to clinical outcomes.

### 3.9. How long should a catheter stay in place for?

The duration for which a drainage catheter must remain in place is dependent on the progress of abscess resolution; however, there is not an exact guideline for how long a drainage catheter should reside for. A common notion is that catheter removal can occur when: the drainage output is reduced to 10 to 20 mL/day or less, the size of the abscess cavity is reduced, abscess-related fistulas are absent or resolved, and the patient’s vital signs return to normal levels.^[49]^ To assess the extent of resolution of an abscess, the presence of potential fistulae post-drainage, and whether a catheter is properly functioning, fluoroscopically guided x-ray procedures, or imaging with CT or ultrasound may be performed.^[50]^ The usage of ultrasound or abscessograms to determine if drainage catheters should be removed has been associated with reduced abscess recurrence rates.^[51]^ Removing the catheter before the abscess has been completely drained can lead to recurrence of the fluid collection, but leaving the catheter in for extended durations may cause skin infection at the catheter site.^[4]^ Other drainage catheter complications include hemorrhage, sepsis, and bacteremia.^[52]^ To circumvent poor drainage and completely drain an abscess, a mixture of tissue plasminogen activator diluted in normal saline can be administered into the catheter, followed by clamping the catheter for 30 minutes.^[52]^ Regular monitoring of the patient, the fluid drainage output, and obtaining the necessary follow-up tests can help minimize complications and identify modifications that may need to be made.

### 3.10. When to treat an abscess with antibiotics vs catheter placement?

Despite the prevalence of PAD procedures, there is no current standardized criteria in the literature that defines the necessity of the drainage of bacterial abscesses.^[53]^ This is changing as more studies explore the topic and provide a body of evidence to better understand when the procedure can be performed at the patient’s benefit, rather than relying solely on the prescription of antibiotics. For instance, Hope et al documented a 1 hundred percent success rate of treating “small” (defined as < 3 cm) hepatic abscesses with antibiotics alone. Additionally, they found that the addition of percutaneous abscess drainage to an antibiotic regimen was highly successful for “large” (>3 cm) unilocular hepatic abscesses, but surgical drainage was the preferred method for large, multilocular hepatic abscesses.^[54]^ A literature review of the treatment of abscesses concluded that an abscess diameter of ≥ 5 cm was correlated with a less favorable outcome of antibiotic therapy alone, suggesting that antibiotic therapy remains highly successful until a cutoff size of potentially 5 cm.^[55]^ This evidence does not necessarily correlate to other abscess locations however, which would leave many patients’ abscess treatments up to the judgement of their physicians. Solomkin et al describes an abscess drainage guideline that patients with minimal physiological derangement and a well-circumscribed focus of infection may be treated with antibiotic therapy alone.^[56]^ This guideline reinforces the literatures’ conclusions that small, well-circumscribed, or unilocular abscesses may be a candidate for lone antibiotic therapy, whereas a larger abscess may be a candidate for drainage. A retrospective study in 2020 concluded that abscesses >0.4 cm in depth from the skin surface may require a drainage procedure.^[57]^ A multicenter randomized control trial from 2017 concluded that antibiotic therapy, in addition to incision and drainage, resulted in better short-term outcomes compared to incision and drainage alone.^[58]^

#### 3.10.1. Emerging technology in PAD

In addition to how imaging has been incorporated into PAD, current technological advancements in AI and biodegradable procedure equipment can be leveraged to further improve efficacy and sustainability.

Under the umbrella term of “AI,” machine learning (ML) is a process by which computational systems are provided information datasets from which they analyze underlying patterns that are then used to generate data based on a user’s pre-defined aims.^[59]^ In the medical community, there has been an increased interest in using AI and ML to aid in multiple aspects of clinical practice, such as decision-making, assistance in interventional procedures, and the diagnosis and detection of diseases.^[60,61]^ Artificial neural networks are a type of ML training model that can be used to identify the best management techniques suitable for individual patients based on their unique risk factors and predicted prognoses by learning from clinical data obtained from patients with similar profiles who underwent abscess drainages at a particular institution in the past.^[62]^ Artificial neural networks have already been shown to help with early detection and identification of intraabdominal abscesses by analyzing a combination of patients’ preoperative CT images, laboratory, and clinical presentation.^[63]^ AI has the potential to be dynamically integrated into the procedural aspect of PAD through projecting optimal trajectories for needle-insertion and drainage from analyzing intraoperatively obtained imaging, thus allowing for increased accuracy and patient safety while minimizing surgical risks and errors. This application has been successfully demonstrated in a recent robot-assisted drainage of multiple brain abscesses.^[64]^ There also exists the possibility of augmenting information gathered from training datasets with virtual reality simulators to project example cases for physician education and training purposes.^[65,66]^ For post-procedural outcomes, ML modeling can help develop algorithms based on pre- and intra-procedural patient characteristics and demographics from both the individual patient and past cases from an institution’s electronic medical record, as well as existing procedural risk stratification tools for a particular procedure to predict a patient’s risk for developing complications and suggest possible prophylactic or treatment measures as indicated.^[67,68]^ The resulting algorithms could be constructed to dynamically update according to changes in the patient’s course of disease and can also aid preoperatively in the selection of the most appropriate catheter and technique based on the features we described in this paper. In the process of developing these algorithms, it may also be possible to form standardized clinical criteria for determining how abdominal abscesses should be managed based on patient characteristics.

Based on the literature review, there are currently no described cases of AI-integration in the management of abdominal abscesses. This may change soon, given the current landscape of rising interest in the potential of AI and ML in medicine and continually updating acceptance of AI/ML-associated medical devices. However, it is important to note that there are several challenges in utilizing AI as described above. With regards to the development of AI-tools or algorithms, large datasets are required to ensure that the model generates consistent predictions and avoids bias.^[69,70]^ To obtain these datasets, there needs to be a protected, confidential, and ethical method of sharing patient records with patient consent, as well as health record information from databases at an inter-institutional, national, and possibly global level. Although the HIPAA Privacy Rule outlines the federal standards of using patient medical information for the US, it is difficult to reconcile differences among international guidelines. Therefore, there is a concern of maintaining stewardship and protecting obtained patient information.^[71]^ Afterwards, the developed AI/ML models need to be validated across multiple institutions to guarantee efficacy in practical application, as well as troubleshoot any issues that may arise during the model’s execution. This entire process may necessitate a long developmental period before an AI/ML model can be released for widespread use.

### 3.11. Key takeaways and what should future research explore?

This comprehensive review highlights several important recommendations that physicians should be aware of regarding PAD. First, the Seldinger technique is preferred over the Trocar technique in most cases due to decreased patient pain and morbidity and less risk of adjacent organ damage and hemorrhage. Second, choosing what size catheter to use for PAD is highly physician dependent and depends on several factors, such as abscess size and viscosity of abscess. Our literature findings suggest that practitioners should try to place the smallest sized catheter they can, such as an 8F to 10F, to achieve a successful outcome as larger sized catheters, 12F to 14F, increase patient discomfort, procedure time, the risk of complication and bleeding; Third, when deciding on the correct time to remove a drain, it is important to properly evaluate the patient as opposed to just relying on a specific time frame. Important things to evaluate include abscess resolution, which can be obtained using fluoroscopically guided x-ray procedures, or imaging with CT or ultrasound, and fluid drainage output. For the latter, it is recommended that the catheter can be removed when the drainage output is reduced to 10 to 20 mL/d or less. Lastly, the decision to use or not to use antibiotic therapy seems to depend on 4 things, size of abscess, location of abscess, depth of abscess from skin, and physician preference. As a rule of thumb, most abscesses < 3 cm can most likely be treated with antibiotics alone, while larger abscesses may necessitate the need for drain placement. Abscesses < 0.4 cm from the skin’s surface will likely not require a drain and can likely be treated with antibiotics alone. When placing a drain for larger abscesses, adding an antibiotic regimen as well will likely result in better outcomes compared to drain placement alone.

As shown in our review, there are still many aspects of this PAD that can be improved upon. The current design of commercially available catheters can be improved upon by decreasing the number of side holes since most flow is seen in the proximal side holes; as such, we should start trying to design catheters such as this way described. How long a catheter should stay in place for after placement, and factors that affect this duration, is an area of interest that future studies should endeavor as there is a lack of strict guidelines on when a catheter should be removed as mentioned previously. Other areas worth investigating include what size catheter should be used for various abscess sizes and when administering antibiotics alone can potentially produce a better triumph compared to placement of a drainage catheter. AI-integration in the management of abdominal abscesses currently does not exist; however, given the increased interest and potential of AI and ML in medicine, this may change soon.

## 4. Conclusion

In this manuscript, we reviewed the history of catheter use and various aspects of PAD, including common imaging modalities used, catheter anatomy, how a catheter is placed, what size catheter to use, the quantity of side holes in catheters, length of catheter stay, and usage of antibiotics, to find the most efficient design and plan for optimizing clinical success when performing PAD procedures. We suggest that practitioners should try to place the smallest sized catheter they can to minimize patient discomfort, procedure time, and risk of complications, and the primary method used for deploying a catheter should be Seldinger. In addition, before removing a drainage catheter, practitioners should thoroughly evaluate the patient by looking at fluid drainage output and obtaining imaging to assess abscess resolution. Lastly, the decision to use or not to use antibiotic therapy seems to depend on multiple entities. A good rule of thumb though is that most abscesses < 3 cm in size and/or abscesses < 0.4 cm from the skin’s surface will likely not require a drain and can likely be treated with antibiotics alone. When placing a drain for larger abscesses, adding an antibiotic regimen will likely yield better outcomes.

## Acknowledgments

The authors would like to thank Dr Johan Mattelaer for giving us permission to include his image in this manuscript.

## Author contributions

**Conceptualization:** Christopher Stevens.

**Investigation:** Christopher Stevens, Dylan Scott, Prerana Ramesh, Amanda Ragland, Coplen Johnson, Joshua Strobel, Kevin Malone, Horacio D’Agostino, Chaitanya Ahuja, Luis De Alba.

**Methodology:** Christopher Stevens, Luis De Alba.

**Project administration:** Christopher Stevens.

**Resources:** Christopher Stevens, Dylan Scott, Prerana Ramesh, Amanda Ragland, Coplen Johnson, Joshua Strobel, Kevin Malone, Horacio D’Agostino, Chaitanya Ahuja, Luis De Alba.

**Supervision:** Luis De Alba.

**Visualization:** Christopher Stevens, Dylan Scott, Prerana Ramesh, Amanda Ragland, Coplen Johnson, Joshua Strobel, Kevin Malone, Horacio D’Agostino, Chaitanya Ahuja, Luis De Alba.

**Writing – original draft:** Christopher Stevens, Dylan Scott, Prerana Ramesh, Amanda Ragland, Coplen Johnson, Joshua Strobel, Kevin Malone, Horacio D’Agostino, Chaitanya Ahuja, Luis De Alba.

**Writing – review & editing:** Christopher Stevens, Dylan Scott, Prerana Ramesh, Amanda Ragland, Coplen Johnson, Joshua Strobel, Kevin Malone, Horacio D’Agostino, Chaitanya Ahuja, Luis De Alba.

## References

[1] Bowman JK. Abscess incision and drainage. Prim Care. 2022;49:39–45. 35125157 10.1016/j.pop.2021.10.002

[2] De Filippo M Puglisi S D’Amuri F. CT-guided percutaneous drainage of abdominopelvic collections: a pictorial essay. Radiol Med. 2021;126:1561–70. 34415507 10.1007/s11547-021-01406-z PMC8702416

[3] De Filippo M Puglisi S D’Amuri F. CT-guided percutaneous drainage of abdominopelvic collections: a pictorial essay. Radiol Med. 2021;126:1561–70. 34415507 10.1007/s11547-021-01406-z PMC8702416

[4] Harclerode TP Gnugnoli DM. Percutaneous Abscess Drainage. StatPearls; 2023. 33232026

[5] Tan SY Poole PS. Allan MacLeod Cormack (1924-1998): discoverer of computerised axial tomography. Singapore Med J. 2020;61:4–5 32043158 10.11622/smedj.2020003 PMC7900806

[6] Cormack AM. Representation of a function by its line integrals, with some radiological applications. J Appl Phys. 1963;34:2722–7.

[7] Cormack AM. Representation of a function by its line integrals, with some radiological applications. II. J Appl Phys. 1964;35:2908–13.

[8] Bhidé A Datar S Stebbins K. Case histories of significant medical advances: development of computed tomography. Harvard Business School: Accounting & Management Unit Research Paper Series; 2019.

[9] Shorvon SD. A history of neuroimaging in epilepsy 1909–2009. Epilepsia. 2009;50(s3):39–49 19298431 10.1111/j.1528-1167.2009.02038.x

[10] Beckmann EC. CT scanning the early days. Br J Radiol. 2006;79:5–8. 16421398 10.1259/bjr/29444122

[11] Pelc NJ. Recent and future directions in CT imaging. Ann Biomed Eng. 2014;42:260–8. 24435658 10.1007/s10439-014-0974-z PMC3958932

[12] Jensen JA. Medical ultrasound imaging. Prog Biophys Mol Biol. 2007;93:153–65 17092547 10.1016/j.pbiomolbio.2006.07.025

[13] Sahlani L Thompson L Vira A Panchal AR. Bedside ultrasound procedures: musculoskeletal and non-musculoskeletal. Eur J Trauma Emerg Surg. 2016;42:127–38. 26059560 10.1007/s00068-015-0539-3

[14] Griffin DR. Return to the magic well: echolocation behavior of bats and responses of insect prey. BioScience. 2001;51:555–6.

[15] Kane D Grassi W Sturrock R Balint PV. A brief history of musculoskeletal ultrasound: “From bats and ships to babies and hips.”. Rheumatology (Oxford). 2004;43:931–3. 15213339 10.1093/rheumatology/keh004

[16] Doppler C. Ueber das farbige Licht der Doppelsterne und einiger anderer Gestirne des Himmels: Versuch einer das Bradley’sche Aberrations-Theorem als integrirenden Theil in sich schliessenden allgemeineren Theorie. K. Böhm Gesellschaft der Wissenschaften; 1903.

[17] Kaproth-Joslin KA Nicola R Dogra VS. The history of us: from bats and boats to the bedside and beyond: RSNA centennial article. Radiographics. 2015;35:960–70. 25822324 10.1148/rg.2015140300

[18] Zimmerman D. Paul Langevin and the discovery of active sonar or asdic. Le Marin du Nord. 2002;12:39–52

[19] Dussik KT. Über die Möglichkeit, hochfrequente mechanische Schwingungen als diagnostisches Hilfsmittel zu verwerten. Z ges Neurol Psychiatr, 1942;174:153–68.

[20] Clark RA Towbin R. Abscess drainage with CT and ultrasound guidance. Radiol Clin North Am. 1983;21:445–59. 6356216

[21] Kobayashi SD Malachowa N DeLeo FR. Pathogenesis of staphylococcus aureus abscesses. Am J Pathol. 2015;185:1518–27. 25749135 10.1016/j.ajpath.2014.11.030 PMC4450319

[22] Stevens CM Malone K Champaneri D Gavin N Harper D. A primer and literature review on internal and external retention mechanisms for catheter fixation. Cureus. 2022;14:e24616. 35664377 10.7759/cureus.24616 PMC9150508

[23] Feneley RC Hopley IB Wells PN. Urinary catheters: history, current status, adverse events and research agenda. J Med Eng Technol. 2015;39:459–70. 26383168 10.3109/03091902.2015.1085600 PMC4673556

[24] Surgical instruments in Greek and Roman times. Nature. 1907;76:468–469.

[25] Hippocrates, AFMAOMBC. The Genuine Works of Hippocrates. Sydenham Society; 1849.

[26] Mattelaer JJ Billiet I. Catheters and sounds: the history of bladder catheterisation. Paraplegia. 1995;33:429–33. 7478735 10.1038/sc.1995.95

[27] Poirier J. Jean-Baptiste Vincent Laborde (1830-1903), forgotten neurologist and neurophysiologist. Geriatr Psychol Neuropsychiatr Vieil. 2015;13:73–82. 25786426 10.1684/pnv.2015.0518

[28] Willette PA Coffield S. Current trends in the management of difficult urinary catheterizations. West J Emerg Med. 2012;13:472–8. 23359117 10.5811/westjem.2011.11.6810 PMC3555603

[29] Mueller PR. The evolution of image-guided percutaneous abscess drainage: a short history. Semin Interv Radiol. 2003;20:171–6.

[30] Mc FA Chang KP Wong CC. Solitary pyogenic abscess of the liver treated by closed aspiration and antibiotics; a report of 14 consecutive cases with recovery. Br J Surg. 1953;41:141–52. 13093985 10.1002/bjs.18004116606

[31] Doust BD Quiroz F Stewart JM. Ultrasonic distinction of abscesses from other intra-abdominal fluid collections. Radiology. 1977;125:213–8. 897172 10.1148/125.1.213

[32] Gerzof SG Robbins AH Birkett DH Johnson WC Pugatch RD Vincent ME. Percutaneous catheter drainage of abdominal abscesses guided by ultrasound and computed tomography. AJR Am J Roentgenol. 1979;133:1–8. 110038 10.2214/ajr.133.1.1

[33] Haaga JR Alfidi RJ Havrilla TR. CT detection and aspiration of abdominal abscesses. AJR Am J Roentgenol. 1977;128:465–74. 402843 10.2214/ajr.128.3.465

[34] Karlson KB Martin EC Fankuchen EI. Non-surgical drainage of intra-abdominal and mediastinal abscesses: a report of twelve cases. Cardiovasc Intervent Radiol. 1981;4:170–6. 7285054 10.1007/BF02552419

[35] Martin EC Fankuchen EI Neff RA. Percutaneous drainage of abscesses: a report of 100 patients. Clin Radiol. 1984;35:9–11. 6690189 10.1016/s0009-9260(84)80216-0

[36] vanSonnenberg E Wing VW Casola G. Temporizing effect of percutaneous drainage of complicated abscesses in critically ill patients. AJR Am J Roentgenol. 1984;142:821–6. 6199965 10.2214/ajr.142.4.821

[37] Segal A. Charriere’s scale. Hist Sci Med. 2016;50:257–62. 30005449

[38] Seldinger SI. Catheter replacement of the needle in percutaneous arteriography; a new technique. Acta Radiol. 1953;39:368–76. 13057644 10.3109/00016925309136722

[39] Higgs ZC Macafee DAL Braithwaite BD Maxwell-Armstrong CA. The Seldinger technique: 50 years on. Lancet. 2005;366:1407–9. 16226619 10.1016/S0140-6736(05)66878-X

[40] Charles HW. Abscess drainage. Semin Intervent Radiol. 2012;29:325–36. 24293807 10.1055/s-0032-1330068 PMC3577622

[41] Ballard DH Alexander JS Weisman JA Orchard MA Williams JT D’Agostino HB. Number and location of drainage catheter side holes: in vitro evaluation. Clin Radiol. 2015;70:974–80. 26084555 10.1016/j.crad.2015.05.004

[42] Schwartz L Vaughan-Neil E. Comparison of the performance of pigtail catheters with different number of sideholes. Cathet Cardiovasc Diagn. 1977;3:421–4. 603910 10.1002/ccd.1810030413

[43] Bishawi M.., Drainage performance of a novel catheter designed to reduce drainage catheter failure. J Clin Interv Radiol ISVIR. 2020;4:9–15.

[44] Pope MC Ballard DH Sticker AL Adams S Ahuja C D’Agostino HB. Fluid flow patterns through drainage catheters: clinical observations in 99 patients. J La State Med Soc. 2018;170:146–50. 30686841 PMC6347390

[45] Commander CW Wilson SB Bilaj F Isaacson AJ Burke CT Yu H. CT-guided percutaneous drainage catheter placement in the abdomen and pelvis: predictors of outcome and protocol for follow-up. J Vasc Interv Radiol. 2020;31:667–73. 32113797 10.1016/j.jvir.2019.09.026

[46] Men S Akhan O Koroglu M. Percutaneous drainage of abdominal abcess. Eur J Radiol. 2002;43:204–18. 12204403 10.1016/s0720-048x(02)00156-0

[47] Celik E Goertz L Henze J. Evaluation of viscosities of typical drainage fluids to promote more evidence-based catheter size selection. Sci Rep. 2023;13:22178. 38092810 10.1038/s41598-023-49160-8 PMC10719316

[48] Park JK Kraus FC Haaga JR. Fluid flow during percutaneous drainage procedures: an in vitro study of the effects of fluid viscosity, catheter size, and adjunctive urokinase. AJR Am J Roentgenol. 1993;160:165–9. 8416618 10.2214/ajr.160.1.8416618

[49] Robert Lewandowski LM Patel P Kandarpa K. Kandarpa Handbook of Interventional Radiologic Procedures. Lippincott Williams & Wilkins; 2023.

[50] Radiology, A.C.o. ACR–SIR–SPR practice parameter for specifications and performance of image-guided drainage/aspiration of abscesses and fluid collections. 2023. https://www.acr.org/-/media/ACR/Files/Practice-Parameters/PDFAC.pdf.

[51] Springer JE Doumouras AG Nair S Eskicioglu C Forbes S. Does imaging before percutaneous drain removal affect rates of intra-abdominal abscess recurrence? J Surg Res. 2018;232:408–14. 30463749 10.1016/j.jss.2018.06.062

[52] Dariushnia SR Mitchell JW Chaudry G Hogan MJ. Society of interventional radiology quality improvement standards for image-guided percutaneous drainage and aspiration of abscesses and fluid collections. J Vasc Interv Radiol. 2020;31:662–6.e4. 32061521 10.1016/j.jvir.2019.12.001

[53] Bassetti M Eckmann C Giacobbe DR Sartelli M Montravers P. Post-operative abdominal infections: epidemiology, operational definitions, and outcomes. Intensive Care Med. 2020;46:163–72. 31701205 10.1007/s00134-019-05841-5

[54] Hope WW Vrochides DV Newcomb WL Mayo-Smith WW Iannitti DA. Optimal treatment of hepatic abscess. Am Surg. 2008;74:178–82. 18306874

[55] Bamberger DM. Outcome of medical treatment of bacterial abscesses without therapeutic drainage: review of cases reported in the literature. Clin Infect Dis. 1996;23:592–603. 8879785 10.1093/clind/23.1.592

[56] Solomkin JS Mazuski JE Bradley JS. Diagnosis and management of complicated intra-abdominal infection in adults and children: guidelines by the Surgical Infection Society and the Infectious Diseases Society of America. Surg Infect (Larchmt). 2010;11:79–109. 20163262 10.1089/sur.2009.9930

[57] Russell FM Rutz M Rood LK McGee J Sarmiento EJ. Abscess size and depth on ultrasound and association with treatment failure without drainage. West J Emerg Med. 2020;21:336–42. 32191191 10.5811/westjem.2019.12.41921 PMC7081847

[58] Daum RS Miller LG Immergluck L; DMID 07-0051 Team. A placebo-controlled trial of antibiotics for smaller skin abscesses. N Engl J Med. 2017;376:2545–55. 28657870 10.1056/NEJMoa1607033 PMC6886470

[59] Sun X. Supervised machine learning for exploratory analysis in family research. J Marriage Fam. 2024;86:1468–94.

[60] Sardar P Abbott JD Kundu A Aronow HD Granada JF Giri J. Impact of artificial intelligence on interventional cardiology: from decision-making aid to advanced interventional procedure assistance. JACC Cardiovasc Interv. 2019;12:1293–303. 31320024 10.1016/j.jcin.2019.04.048

[61] Kumar K Jairam K Ambedkar C. An in-depth study of machine learning in artificial intelligence. Educ Adm Theory Pract. 2023;29:2401–8.

[62] Wilson R Bettis-Outland H, Can artificial neural network models be used to improve the analysis of B2B marketing research data? J Bus Ind Mark. 2019;35:495–507.

[63] Freed KS Lo JY Baker JA. Predictive model for the diagnosis of intraabdominal abscess. Acad Radiol. 1998;5:473–9. 9653463 10.1016/s1076-6332(98)80187-6

[64] Tabourel G Le Turnier P Buffenoir K Roualdes V. Robot-assisted stereotactic multiple brain abscesses’ puncture: technical case report. Acta Neurochir (Wien). 2022;164:845–51. 34410501 10.1007/s00701-021-04955-4

[65] Mao RQ Lan L Kay J. Immersive virtual reality for surgical training: a systematic review. J Surg Res. 2021;268:40–58. 34284320 10.1016/j.jss.2021.06.045

[66] Bernier GV Sanchez JE. Surgical simulation: the value of individualization. Surg Endosc. 2016;30:3191–7. 27338581 10.1007/s00464-016-5021-8

[67] Mohammadi I Rajai Firouzabadi S Hosseinpour M. Using artificial intelligence to predict post-operative outcomes in congenital heart surgeries: a systematic review. BMC Cardiovasc Disord. 2024;24:718. 39702050 10.1186/s12872-024-04336-6 PMC11660586

[68] Balch JA Ruppert MM Shickel B. Building an automated, machine learning-enabled platform for predicting post-operative complications. Physiol Meas. 2023;44:024001. 10.1088/1361-6579/acb4db PMC9910093 36657179

[69] Long B Cremat DL Serpa E Qian S Blebea J. Applying artificial intelligence to predict complications after endovascular aneurysm repair. Vasc Endovascular Surg. 2024;58:65–75. 37429299 10.1177/15385744231189024

[70] Hashimoto DA Rosman G Rus D Meireles OR. Artificial intelligence in surgery: promises and perils. Ann Surg. 2018;268:70–6. 29389679 10.1097/SLA.0000000000002693 PMC5995666

[71] Kerr RS. Surgery in the 2020s: implications of advancing technology for patients and the workforce. Future Healthc J. 2020;7:46–9. 32104765 10.7861/fhj.2020-0001 PMC7032584

